# Review of novel energetic polymers and binders – high energy propellant ingredients for the new space race

**DOI:** 10.1080/15685551.2019.1575652

**Published:** 2019-03-01

**Authors:** Tianze Cheng

**Affiliations:** Pitzer College, Claremont, CA, USA

**Keywords:** Energetic-binder, azide, propellant, solid-propellant, energetic-materials, specific-impulse, mechanical-properties, HTPB

## Abstract

Current solid rocket propellant formulations still employ traditional ingredients utilized since the 1960s, such as hydroxyl terminated polybutadiene (HTPB). Recent advances in energetic polymer see many binders capable of providing higher specific impulse and burn rates over HTPB. As shown by calculations, even slight increases in specific impulse can drastically increase the maximum payload of a launch system. Therefore, replacing HTPB with energetic binders could provide heavy space missions the needed extra boost. Energetic binders could also be paired with chlorine-free energetic oxidizers to synergistically provide a specific impulse exceedingly higher than the current formulation while reducing pollution. A comprehensive evaluation of the synthesis, mechanical properties, and performance of various trending and overlooked energetic polymers is described. Several outstanding candidates show promising properties to replace HTPB.

## 1. Introduction

Solid propulsion is widely used in space operations and its mixture is usually composed of a polymeric binder, metallic fuel, and oxidizer [1–3]. As shown by calculations, a mere 5% increase in specific impulse can increase the range of an intercontinental ballistic missile by a remarkable 45% [4]. Therefore, increasing the specific impulse of current propellant formulations is strongly desired.

Since most other methods of propulsion, such as nuclear thermal rocket, plasma propulsion, hall-effect thrusters, VASIMR, and other variations of ion propulsion, have many limitations and only see potential in outer-space applications [5]. It appears that improving the current formulations of chemical propulsions might be the optimal strategy to launch hefty payloads amidst the thriving space industry [6].

**Figure 1.**
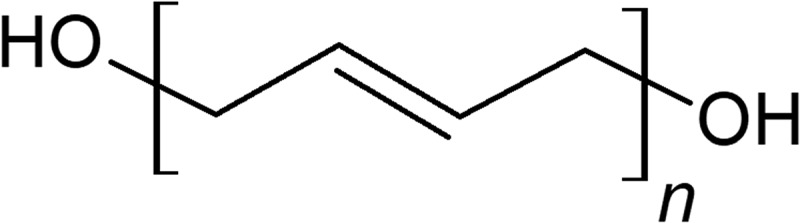
HTPB.

Most current solid propellant formulation popularly utilizes hydroxyl terminated polybutadiene (HTPB) as its binder (Figure 1). The binder component of a propellant not only hold together the entire mixture as a solid component but also serves as a combustible fuel [1–3]. Usually, the binder is an inert component of the solid propellant that does not contribute to the overall propellant specific impulse. Although the carbon backbone of the polymer HTPB is similar to that of a hydrocarbon which is combustible, many emerging energetic binders has shown promise to replace HTPB in this regard to provide higher specific impulse. In this paper, a list of currently trending and overlooked energetic binders, including their synthesis, properties, feasibility, and performance, will be discussed.

Since aerospace companies and space missions tend to lay toward low-risk options, the transition from using APCP to another propellant will indeed require some courage. Although HTPB offers reliable mechanical properties, its carbon backbone offers no energetic properties that positively contribute to specific impulse while also significantly dampening the oxygen balance of the composition [7]. Energetic polymers not only can increase specific impulse, but also increase burn rate, density, and combustion temperature [8–10].

## 2. Azido polymers

Advances in the energetic polymers and plasticizers in the past several decades saw few candidates with exceptional properties, such as glycidyl azide polymer (GAP). GAP was first synthesized in 1972 by Vandenburg from the reaction of sodium azide and polyepichlorohydrin (PECH) in dimethylformamide (DMF) [11]. The potential of GAP was discovered in the early 80s, research and studies in stability, curing methods, compatibility has since been trending [12].

Synthesis of GAP starts from a diol or triol such as butanediol or glycerin, then reacting with epichlorohydrin in an organic solvent to form PECH [13]. PECH is then mixed with sodium azide to result in GAP and sodium chloride, in organic solvents such as DMF [14–16]. The resulting polymer, GAP, is energetic with a high burn rate owing to the exothermic decomposition of the azide group, which also is the reason of the high enthalpy of formation of the polymer: +957 kJ/kg [17,18].

What is more, GAP has been synthesized into copolymers with other energetic binders. Some notable copolymers include using poly(bis(azidomethyl)oxetane), also known as Poly-BAMO [19]. BAMO-GAP-BAMO blocks could be produced via one step azidation of BCMO-PECH-BCMO polymer. Poly-BAMO itself has attracted interest due to its higher azide content than GAP. BAMO was found to be compatible with other energetic materials such as CL-20, HMX, and RDX [19–20]. Unfortunately, GAP and BAMO have rather poor mechanical properties, GAP’s properties are better.

To solve this problem, grafting GAP onto HTPB, in GAP-HTPB copolymers. or by using polybutadiene as the starting diol of GAP synthesis to produce GAP-PB-GAP copolymer, resulted in polymers with improved properties [21,22]. However, long chain of polybutadiene means less azido polymer can be grafted on to the polymer before reaching the upper molecular limit for desired properties, which hinders the energy content and defeats the purpose of energetic polymers.

Despite a significant amount of research devoted toward GAP-based copolymers, breakthrough improvements of GAP’s mechanical properties applied other strategies. Many studies and investigations have investigated isocyanate-free curing of GAP by using polyfunctional alkynes and have provided significantly better mechanical properties than urethane cured. Azido groups from GAP react with polyfunctional alkynes to yield 1,2,3-triazoles. GAP cured with bis-propargyl succinate (BPS) has shown better mechanical properties to diisocyanate cured urethane counterpart [21–29].

Several triazoles cured polymer suffers from various degrees of swelling and may require a careful mixture of ratios and other solutions [23–27]. A recent study cured poly-butadiene with GAP using Propargyl terminated polybutadiene(PTPB) in a 2:1 ratio, respectively. This polymer has better mechanical properties than the urethane cured GAP-PB-GAP copolymer as shown in Table 1. PTPB-GAP binder shows promising properties with good mechanical properties and glass transition temperature [27]. Other groups have developed an alternative to BPS in the triazole curing of GAP using Bis-propargyl hydroquinone(BPHQ) and dimethyl 2,2-di(prop-2-ynyl)malonate(DDPM) and appear to show better mechanical properties than the BPS cured formulation [25,28].

**Table 1.** Mechanical properties of glycidyl azide polymer (GAP) – A comparison of mechanical properties between urethane and triazole cured GAP. For reference, HTPB-based APCP has a tensile strength of 0.7 N mm-2 and elongation maximum of around 60–80%.

Polymer Mixture Formulation by part:/*Type of curing*	Tensile Strength (N mm^−2^)	Shore A Surface Hardness	Elongation Maximum (%)	Elongation at Break (%)	E Modulus (N mm^−2^)	Glass Transition Temp (°C)	References
GAP-PB-GAP 1:1 TDI/TMP/Urethane	1.7	9	-	110	-	−59	[21]
GAP-PB-GAP 1:1 TDI/TMP/Urethane	0.4	26	-	120	-	−59	[21]
GAP-PB-GAP copolymer/Urethane	0.3	-	13	14	0.9	-	[22]
GAP 93:7 BPS./Triazole	0.1	34	82	82	0.2	−38	[23]
GAP 91.4: 8.6 BPS./Triazole	0.2	58	57	57	0.6	−34	[23]
GAP N100/Urethane*	0.3	14	16	21	5.4	−44	[24]
GAP BPS/Triazole*	0.3	66	13	15	3.0	−42	[24]
GAP 1:1 BPHQ/Triazole	0.1	-	65	73	0.2	−32	[25]
GAP 1.4:1 BPHQ/Triazole	0.2	-	26	27	0.7	-	[25]
GAP 2.5:1BPHQ./Triazole	1	-	28	28	4.2	-	[25]
GAP 13.2:1 BPS/Triazole	0.1	9	82	82	0.2	−37	[23]
GAP 10.5:1 BPS/Triazole	0.2	26	57	57	0.6	−34	[23]
GAP Triol 12:3 BPS/Triazole	0.2	22	57	57	0.4	−36	[23]
GAP 6.8:1 N100/Urethane*	0.3	-	10.5	19	4.7	−55	[26]
GAP 1.4:1 PTPB/Triazole	0.8	-	-	73	1.9	−82	[27]
GAP 16.3:1 DDPM/Triazole	0.2	-	-	50	~2	−44	[28]
GAP 5.5:1 DDPM/Triazole	4.5	-	-	80	~9	−34	[28]
GAP 3.3:1 DDPM/Triazole	13.2	-	-	30	174	−5	[28]

Dashed cells represent no data or not reported, *The following formulations contain other solid mixtures or plasticizers that may shift the properties of the resulting polymer.

**Figure 2.**
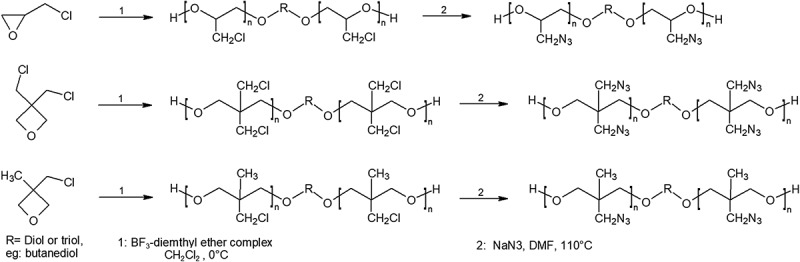
Synthesis of GAP (top), Poly-BAMO (middle), and Poly-AMMO (bottom) as described above.

Recently a novel synthesis of GAP and poly (3-azidomethyl-3-methyl oxetane) (poly-AMMO) from mesylate polymeric precursors were developed [16]. Usually, the azide group is added on to an energetic polymer through substitution of a halogen or tosyl group. While halogen could result in incomplete substitutions and the high molecular weight of tosyl group could hinder polymer reactivity as well as cause industrial scale inconvenience. Mesyl chloride shows to be a viable option for industrial scale GAP synthesis. Another synthesis of GAP with microwave assistance has also reported higher purity and complete conversion of chloride to azide during the azidation process of PECH at a lower temperature, as shown in Figure 2 [27].

**Figure 3.**
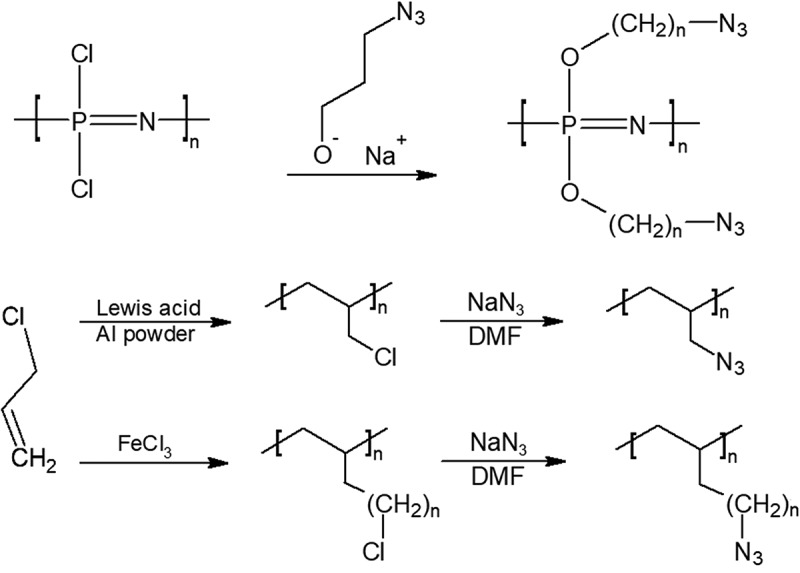
Synthesis of poly(allyl azide) PAA (top), PZ-23, and PZ-24 (bottom). Synthesis of poly(allyl azide) PAA (right), PZ-23 and PZ-24 (left).

Poly(allyl azide) (PAA), a brown colored resin, has been synthesized through the polymerization of allyl chloride followed by reaction with sodium azide to form PAA [29,30]. This route of synthesis , as shown in Figure 3, produced an azide content of around 35–40% which was lower than the theoretical value. The resulting polymer could be cured with ethylene glycol dimethacrylate over the course of a week at 60°C. The above curing process is exothermic, and the cured polymer often contained moisture. Although PAA shows thermal stability, high azide content, and therefore should deliver exceptional performance, however, the curing of the polymer needs further investigation and improvement [31,32].

Another nitrogen-dense polymer, poly(phosphophazene), has been synthesized from the polymerization of Trichloro(trimethylsilyl)phosphoranimine [33,34]. The phosphazene polymers are promising because of its nitrogen dense backbone, the opportunity to include functional groups mean that it could be further decorated into an extremely energetic polymer with a high enthalpy of formation. The reaction of poly(dichlorophosphazene) with sodium salt of 3-azidopropan-1-ol under 18 hour reflux produces the energetic polymer poly(bis-(3-azidopropan-1-oxy)phosphazene) or PZ-23 (n = 3). Using the 3-azidohexane-1-ol produces a longer chain polymer called PZ-24 (n = 6), however the density and azide content is lower than P-23.

Both procedures yield around 80%. With a nitrogen dense the backbone further enriched by azido groups to the side, both PZ-23 and PZ-24 are highly energetic. PZ-23 is particularly energetic and is over 20% more energy dense compared to GAP. PZ-23 has similar nitrogen content as GAP with a glass transition temperature of −73°C. PZ-24 is a viscous yellow polymer with lower energetic properties than PZ-23 due to the longer carbon chain, but it has a superior glass transition temperature of −100°C.

**Figure 4.**
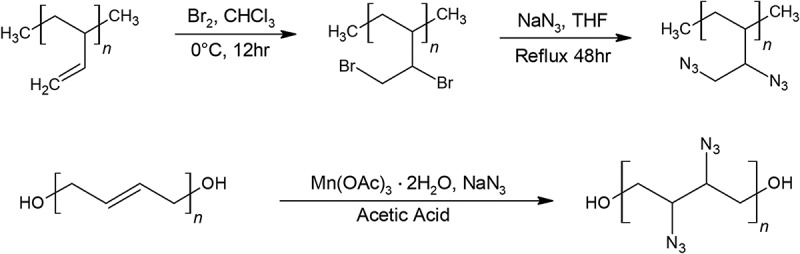
Synthesis of azido-polybutadiene(Azido-PB) (top) and azido-HTPB (bottom).

While HTPB-GAP copolymers do not show significant improvement from GAP, directly adding the energetic azido group onto HTPB has been performed recently by Pant et al. in a 1 step synthesis shown in Figure 4 [35].

This reaction performed in glacial acetic acidic acid at 110°C until the solution turned light yellow, and the azido-HTPB is extracted as a brown viscous liquid. Adding azide groups onto HTPB came at a cost to the properties of HTPB. Merely 15% azidation to the backbone started to notably increase its viscosity and glass transition temperature, and at 20% azidation, azido-HTPB becomes a gelatin-like material. Therefore, around 10% azidation of HTPB results in optimal properties with a low viscosity o 5 Pa·s, similar to that of HTPB’s 11 Pa·s. After 10% azidation to HTPB, the average molecular weight of HTPB increased from 2,550 to 3,050 g/mol which means the resulting azido-HTPB contains about 14% nitrogen by weight. This means 10% azido-HTPB have about half the nitrogen content compared to GAP. The mechanical properties of this otherwise promising polymer have yet to be tested.

The mechanical properties of azido-polybutadiene (azido-PB) and ethylene vinyl acetate (EVA) copolymer binder, however, have been tested by Yoon et al.’s team [36]. This team synthesized azido-PB in a slightly different manner than azido-HTPB, they brominated polybutadiene first, followed by azidation using sodium azide in THF. The result of the copolymer was prepared in 9:1, 8:2, and 7:3 ratio of EVA to azido-PB, respectively, and was found to have decent mechanical properties worth further investigation. Preliminary combustion tests of the copolymer showed that the more azido-PB content, the fiercer the flame twirled, as expected.

## 3. Nitro polymers

**Figure 5.**
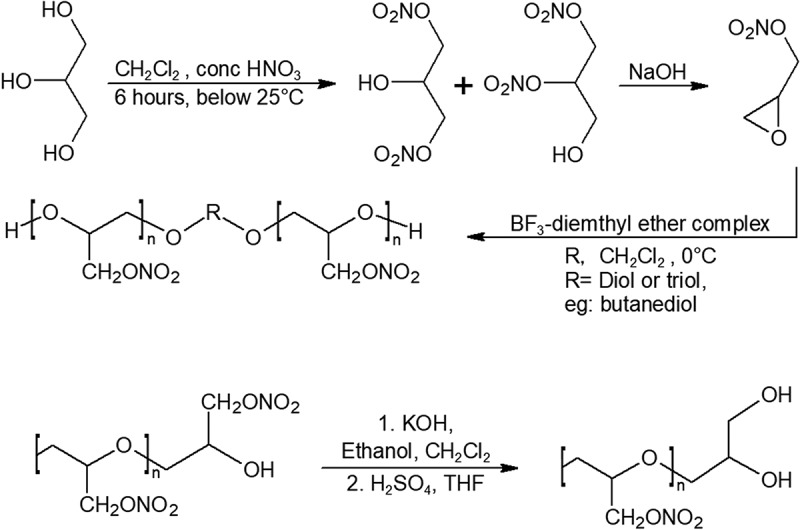
Synthesis of poly-GLYN (top) and end-modified poly-GLYN (bottom).

Nitro groups can contribute to the overall oxygen balance of the propellant formulation when grafted onto the carbon backbone of polymers. The relief in oxygen balance nitro polymers provide means that the oxidizer content could be reduced, and other energetic additives could be added instead. Poly-glycidyl nitrate (poly-GLYN) was one of the promising energetic polymers investigated heavily during the 90s. Unfortunately, when poly-GLYN is cured with isocyanates, it undergoes rapid degradation and gassing due to the low activation energy of its urethane link, making it unviable for almost all applications [37]. Due to the advantages in poly-GLYN’s high-density impulse, oxygen balance, and burn rate, attempts at modifying poly-GLYN’s terminating section was therefore pursued. Diol-terminated and epoxy-terminated poly-GLYN shown in Figure 5 has been synthesized to show long term stability [10,37–40]. One pot modification of poly-GLYN has also been performed by epoxidation of the polymer with potassium hydroxide in a solvent, followed by ring-opening by sulfuric acid [40]. The mechanical properties of end-modified poly-GLYN have not been tested and revealed yet.

**Figure 6.**
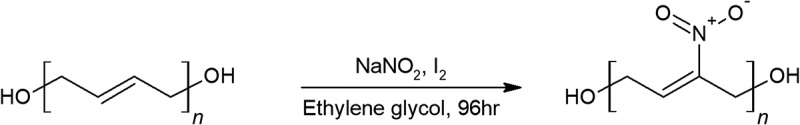
one-pot synthesis of nitro-HTPB.

Nitro-HTPB, or N-HTPB, has been synthesized decades ago. The traditional synthesis required N2O5 to be reacted with HTPB in an inert solvent [41]. This hazardous procedure was performed until a recent single pot synthesis was recently discovered [42]. The one-pot synthesis of N-HTPB, shown in Figure 6, starts with dissolving HTPB and sodium nitrite in ethyl acetate and ethylene glycol, respectively. After mixing the two solutions at 0°C, introduce iodine the reaction flask for 4 days at room temperature. The resulting polymer had about nitro groups grafted onto about 15% of the double bonds on the carbon backbone, increasing the average molecular weight of the polymer from around 2500–3100 gmol^–^^1^ [42].

With the addition of nitro groups, N-HTPB is miscible with energetic plasticizers with superior oxygen balance and burn rate than HTPB. N-HPTB has shown to be thermally stable up to 170°C, and propellants using N-HTPB also shows higher density and burn rate than propellants using HTPB as its binder [10,41–45]. The production cost of end-modified poly-GLYN and poly-NIMMO is rather high. Now with the novel one-step synthesis of N-HTPB, it might be an optimal nitro-polymer candidate for scale up production and further testing.

## 4. Other energetic binders

Recently, one study has shown a solid propellant containing H2O2 cured at room temperature using a novel polymer called LZY as its binder [46]. The propellant contains 98% concentrated H2O2 absorbed by sodium acrylate to form a solid slush, with the addition of stabilizers.

The novel polymer, LZY, is compatible with the highly reactive peroxide oxidizer, but its chemical structure was not revealed. However, the author did state that LZY polymer was made from silicon-containing rubber that had a carbon-carbon backbone without silicon-oxygen bonds. Due to the low amounts of silicon, it also did not interfere with specific impulse. This propellant containing the LZY polymer based solid propellant has a reported specific impulse (Isp) that exceeds APCP by nearly 20 s [46].

**Figure 7.**
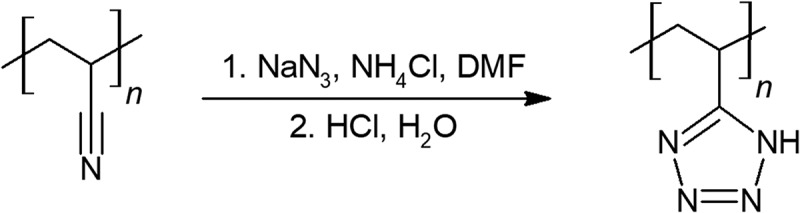
Synthesis of polyvinyl tetrazole.

Polyvinyl tetrazole is synthesized from polyacrylonitrile and sodium azide in a single step shown in Figure 7 [47–49]. Mechanical properties of polyvinyl tetrazole were only simply evaluated. A conversion of 18% of cyano groups to tetrazole provided promising rubber-plastic like properties, however at 43% and higher cyano to tetrazole conversion, the polymer was stiff and brittle.

Polyvinyl tetrazole has also been synthesized in copolymers to produce desired properties, and the tetrazole ring formation may potentially apply to other acrylonitrile containing copolymers, such as the widely used PBAN in the current formulations of solid propellants.

**Figure 8.**
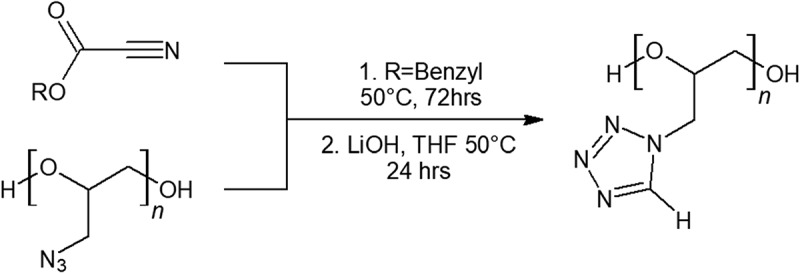
Synthesis of glycidyl tetrazole polymer (GTP).

Aronson has described the synthesis of glycidyl tetrazole polymer (GTP) in a two-step conversion starting from GAP drawn in Figure 8 [50]. Ethyl or benzyl cyanoformates clicks over the azido groups on GAP to form the tetrazole ring after 3 days of reaction time. Decarboxylation of the esters is performed by lithium hydroxide. The complete transition of the azide group from GAP to GTP results in a solid polymer. Although GTP has a higher nitrogen content, GTP has a slightly lower energy than GAP by weight. It is claimed that a balance between improved storage and performance was found between quarter conversion of GAP to GTP [50]. Although GTP did not decompose as exothermically as GAP and may prove to be more stable in storage, the extra synthesis and reduction in performance are undesired. This may mean GTP is not worthy of further investigation, due to the cost of synthesis and lower energy content than its precursor.

**Figure 9.**
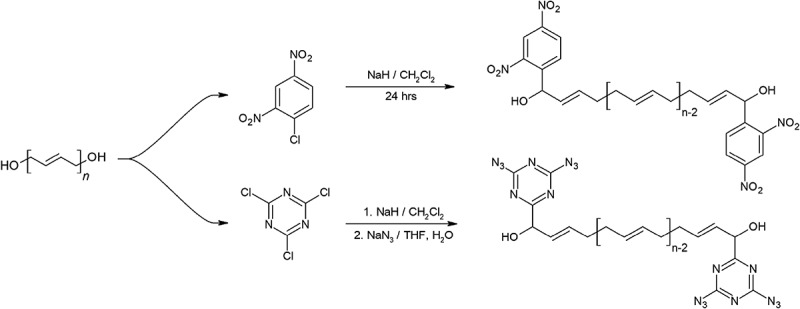
Synthesis of end modified HTPB.

Several terminal modified HTPB has been synthesized by Shankar and his colleagues as shown in Figure 9. The modification of HTPB was performed using 1-chloro-2, 4-dinitrobenzene (DNCB), where the energetic dinitrobenzene group hanged to carbon backbone near the terminating diols, creating HTPB-DNCB. It is synthesized using a 1 step method involving sodium hydride and the DNCB. The resulting HTPB-DNCB have an amber color [51]. This polymer was found to have better mechanical properties than HTPB along with a significant improvement in burn rate over HTPB [52,53]. A static firing test was performed to compare HTPB and HTPB-DNCB-based APCP formulations, with equal amounts of ammonium perchlorate and aluminum powder, the HTPB-DNCB-based APCP produced a specific impulse of 247 compared to 241 to that of HTPB [52]. This difference is further amplified when comparing for density impulse where HTPB-DNCB outperforms HTPB by a wide margin of 24 s.

Using the same method that synthesized HTPB-DNCB, Shankar’s team later synthesized another polymer using 1-chloro-3,5-diazido-2,4,6-triazine. The addition of nitrogen dense energetic groups to each end of HTPB chains resulted in the milky white polymer HTPB-DT. This polymer not only have good mechanical properties, but it also has a lower viscosity during mixing than HTPB-DNCB counterpart [53,54].

**Figure 10.**
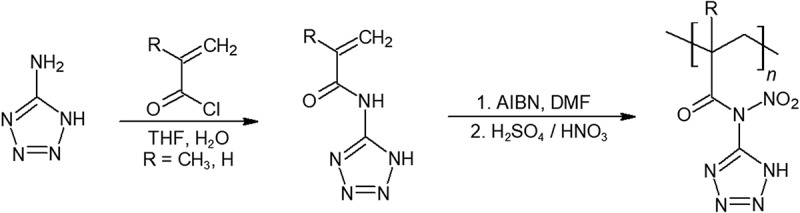
Synthesis of PANT polymer.

Displayed in Figure 10, Betzler et al. have synthesized an impact-insensitive energetic poly-nitrimidotetrazole (PANT) that is completely insensitive to impact with a higher oxygen balance and density, however, the resulting polymer is solid and therefore will likely find limited use [55].

**Figure 11.**
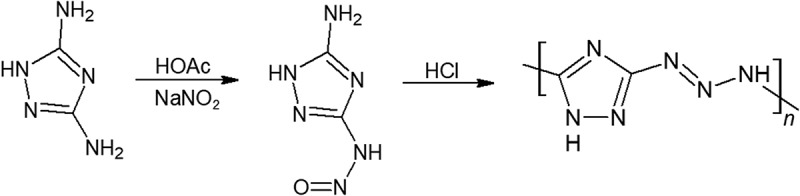
Synthesis of poly-DAT.

Betzler et al. has also synthesized Poly(3-diazoamino-1,2,4-triazole) (Poly-DAT), a novel polymer that contains nearly 80% by weight nitrogen as shown in Figure 11[56]. This polymer is more thermally stable than what it looks like, with a rather high decomposition temperature of 170°C. However, despite its promising energy content, the isolation of this polymer from impurities was difficult. Not only does the diazoamino group react with many reagents, but the polymer also decomposes rather easily. Although poly-DAT does not have satisfactory properties and optimal preparation methods yet, the possibility of a polymer with a nitrogen backbone should be further pursued due to their exceptionally high-energy density. In later calculations, we show that poly-DAT to outperform GAP.

Polytetrahydrofuran (PTHF) based propellant produce promising mechanical properties while having better oxygen balance and higher burn rate than HTPB [57–60]. In fact, in a propellant of 80% ammonium perchlorate to 20% binder by weight mixture, directly replacing HTPB with PTHF results in a 10-second increase in specific impulse. This increase, although less significant than energetic binders, is still quite an improvement considering the low cost and wide availability PTHF.

Although PTHF does not contain any energetic components, it gives a specific impulse comparable to that of energetic polymers due to its higher oxygen balance than HTPB [58–60]. Kohga and his team have been testing various formulations of PTHF and has shown that certain ammonium perchlorate-PTHF-based formulation has nearly twice the burn rate of HTPB-based propellant [57].

The same team also performed various testing of the mechanical properties of PTHF and GPO-based formulations with successful results. It was found that PTHF-based formulations have exceptional mechanical properties that are higher than that of GAP-based formulations [58–60].

The mechanical properties of PTHF and various formulations and molecular weight of glycerol propoxylate (GPO) is given below along with other energetic binder mixtures.

Table 2. Mechanical properties of various energetic polymers. For reference, HTPB-based APCP has a tensile strength of 0.7 N mm-2 and elongation maximum of around 60–80%.

**Table 2.** Mechanical properties of various energetic polymers. For reference, HTPB-based APCP has a tensile strength of around 0.7 N mm^−2^ and elongation maximum of around 60–80%.

Polymer Mixture: Formulation by weight % or by OH:NCO ratio:	Tensile Strength (N mm^−2^)	Shore A Surface Hardness	Elongation Maximum (%)	Elongation at Break (%)	E Modulus (N mm^−2^)	Glass Transition Temp (°C)	References
Azido-PB 2:8 EVA	6.0	-	-	820	7	−34	[36]
Azido-PB 3:7 EVA	5.3	-	-	710	10.8	−38	[36]
Azido-PB	-	-	-	-	-	−59	[36]
HTPB 11% APCP*	0.7	-	74	-	3.0	−66	[39]
Nitro-HTPB 9.1%. APCP*	0.6	-	78	-	2.6	−58	[39]
Nitro-HTPB 12.2%. APCP*	6.7	-	71	-	2.9	−54	[39]
Nitro-HTPB 11%. APCP*	0.8	-	38	-	3.1	−48	[9]
Nitro-HTPB 9.0%. APCP*	0.7	-	45	-	2.8	−52	[9]
HTPB 11%, DOA, IPDI. APCP*	0.7	-	68	-	3.1	−67	[9]
Nitro-HTPB 0.9:1 TDI	0.8	9	-	397	0.2	-	[40]
Nitro-HTPB 1:1 TDI	1.1	19	-	292	0.4	-	[40]
Nitro-HTPB 1.1:1 TDI	1.4	24	-	185	0.7	-	[40]
(Solid H_2_O_2_) SPHP Polymer LZY	0.5	-	-	>100	-	-	[46]
HTPB-CBDT 1:1 IPDI	1.3	-	-	187	0.01	-	[53]
HTPB-CBDT 1:1 2,6-TDI	4.6	-	-	74	0.07	-	[53]
HTPB-DT 1:1 IPDI	2.0	-	-	145	0.02	-	[53]
HTPB-DT 1:1 2,6-TDI	6.4	-	-	93	0.07	-	[53]
650 gmol PTHF 64.1%, Glycerin, IPDI	6.9	-	784	-	-	-	[58]
1400 gmol PTHF 79.4%, Glycerin, IPDI	1.8	-	828	-	-	-	[58]
2900 gmol PTHF 88.9%, Glycerin, IPDI	0.9	-	866	-	-	-	[58]
650 gmol PTHF 31.6%, GPO, IPDI	34	-	60	-	-	-	[59]
650 gmol PTHF 44.4%, GPO, IPDI	17	-	420	-	-	-	[59]
650 gmol PTHF 51.3%, GPO, IPDI	5	-	490	-	-	-	[59]
650 gmol PTHF 65.6%, GPO, IPDI	4	-	940	-	-	-	[59]
1400 gmol PTHF 49.9%, GPO, IPDI	7	-	280	-	-	-	[59]
1400 gmol PTHF 73.0%, GPO, IPDI	2	-	600	-	-	-	[59]

Not reported, *The following formulations contain other solids or plasticizers that may shift the properties of the resulting polymer.

## 5. Propellant performance of energetic binders

Specific impulse was calculated using a highly reliable code developed by the Naval Weapons Center – China Lake, in the late 1970s [61]. When inputting a mixture of propellant ingredients, their density, enthalpy of formation, and quantity, this code is capable of accurately predicting specific impulse, combustion temperature, exhaust gas product, exhaust velocity, and a variety of other propellant characteristics. All properties of energetic polymers are added to the code, density and enthalpy of formation are taken from the previously cited studies. When enthalpy of formation was not available, a semi-empirical and quantum chemistry composite method PM6 and CBS-4M was used to estimate these properties. PM6 was chosen because a similar method, PM3, have been used in energetic materials [62]. However, recently it was shown that the PM6 provided substantially less average error than PM3 [63]. Further calculations were performed using the more accurate composite basis set (CBS-4M) methods, and we found comparable results to that of PM6 [64].

In the calculation, the propellant mixtures have a controlled aluminum content of 16% the total weight. Due to the difference in the oxygen balance between different polymers, the ratio of ammonium perchlorate to energetic binder is added to the code at the optimum ratio for the highest specific impulse. However, some binders are extremely oxygen negative, such as HTPB, and therefore have an extremely high optimal oxidizer to binder ratio. To address the problem and to increase the realism of the calculation, the percentage of total weight of binder had to be at least above 18%, considering for mixing difficulties and the final mechanical properties of the propellant mixture.

Also, in the calculation, azido-HTPB and poly-vinyl tetrazole are of 20% azidation from HTPB and 20% tetrazole transformation from polyacrylamide, respectively. Twenty percent of azide groups were considered as it was pointed out earlier that further azidation starts to significantly worsen the properties of the polymers. GTP is worth no further investigation as it requires an extra synthesis step starting from GAP, while GAP itself has a higher specific impulse.

**Figure 12.**
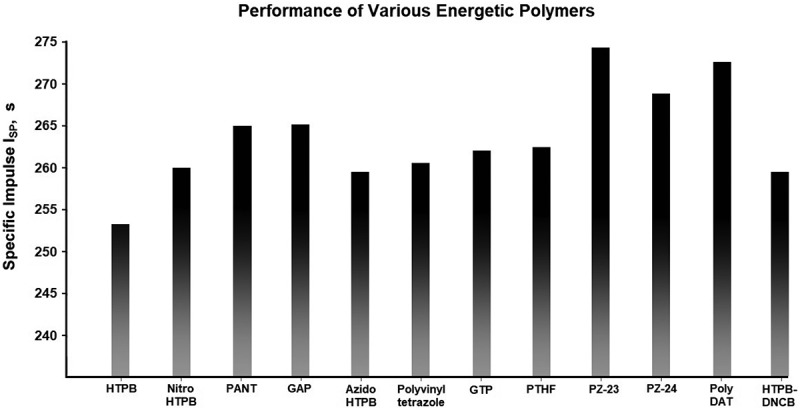
A comparison of performance when swapping out HTPB for energetic polymers in ammonium perchlorate based propellant formulation.

As shown in Figure 12, the increase in specific impulse when replacing HTPB with energetic binders is only limited to around 5–25 seconds of improvement, but we must remember that the binder content is only around a fifth of the propellant’s mixture. Swapping out the polluting ammonium perchlorate for a green energetic oxidizer will synergize with energetic binders to produce a significantly higher specific impulse. In fact, using the same propellant performance evaluation code, an ammonium dinitramide, GAP, and aluminum-based formulation exceeds 284s of specific impulse. This is one of the few currently feasible solid propellant formulations that exceed the performance of the commonly employed liquid oxygen(LOX) and RP1 liquid propellant, which at a 2.4 to 1 ratio of LOX:RP-1, is calculated to have only 281s. Experimentally, SpaceX’s Falcon 9 Merlin 1D engine, which utilizes the same LOX RP-1 formulation, obtains a specific impulse of 282s [65].

## 6. Conclusion & future directions

The energetic nitrogen-rich polymers GAP, poly-phosphazene PZ-23, and Poly-DAT, significantly out-performed HTPB in our calculations. Poly-DAT and PZ-23 exceed the performance of GAP in our calculations. Our computational estimation of PZ-23, as well as the only patent on poly-phosphazene, points toward an extremely high energy content [32,33]. Currently, energetic phosphazene binders are under-researched, and the claimed energy density of PZ-23 if correct, is worth further investigation. As expected, the nitrogen-backbone polymer Poly-DAT also performed well in the calculations due to their exceptionally high heat of formation. Novel nitrogen backbone-based energetic binders also deserve further investigation and exploration.

GAP remains the most well-researched and popular energetic polymer. Its performance significantly exceeds that of HTPB, and new curing methods have shown promising mechanical properties. The two energetic binders HTPB-DNCB and HTPB-TD have significant improved tensile strength, and maximum elongation compared to HTPB and is also worth further testing.

What is more, many novel energetic oxidizers and additives have already been proposed and is undergoing further characterization and development to replace the dirty ammonium perchlorate [66–73]. A combination of energetic oxidizer and energetic binder may push the specific impulse of a propellant mixture to nearly 300s while producing significantly less pollution. In summary, many novel energetic binders also show significant improvements over HTPB in terms of specific impulse with promising emerging data in mechanical properties. The blooming aerospace industry should prepare and open up to the new generation of propellant ingredients surfacing from the current research in energetic materials.
